# Surface Characterization of Cement Paste Exposed to Magnesium Sulfate

**DOI:** 10.3390/ma19173691

**Published:** 2026-08-30

**Authors:** Hani Alanazi

**Affiliations:** 1 Department of Civil and Environmental Engineering, College of Engineering, Majmaah University, Al-Majmaah 11952, Saudi Arabia; hm.alanazi@mu.edu.sa; 2 Engineering Research and Applied Sciences Center, Majmaah University, Al-Majmaah 11952, Saudi Arabia

**Keywords:** cement paste, external sulfate attack, nanoindentation, indentation modulus, microstructural analysis, chemical analysis

## Abstract

Exposure to external sulfate attack can cause significant deterioration of concrete structures. This is a particular concern for concrete structures exposed to sulfate-rich soils, seawater, or groundwater. This study aims to evaluate the impact of external sulfate attack on the surface of cement paste systems. The nanomechanical properties of the surface of the cement paste systems were evaluated using the nanoindentation test. Microstructural and chemical analyses were conducted on the cement paste systems using scanning electron microscopy (SEM) with energy-dispersive X-ray spectroscopy (EDX). Significantly different nanoindentation results were obtained after exposure to magnesium sulfate, coinciding with the development of degraded areas and a Mg-rich surface layer consistent with brucite formation. The microstructural and chemical analyses showed that this brucite layer forms a white layer and covers almost the entire cement paste surface. Based on the nanoindentation results, the volume fraction with an indentation modulus below 9.6 GPa increased from 3.3% before exposure to a MgSO_4_ solution to 8.4% after exposure, due to the degradation of C–S–H and the formation of microcracks. The results of this study revealed that 7 days of exposure to a 2% MgSO_4_ solution was enough to induce noticeable degradation of the cement paste surface. The development of a dense Mg-rich surface layer, consistent with brucite formation, may contribute to reducing ion transport through the outer cement paste region.

## 1. Introduction

Concrete as a construction material has played a significant role in the development of recent modern economies around the world. This is due to the strength and durability of concrete compared to other construction materials such as steel or wood. The availability of raw materials to make concrete made it the preferred construction material in many regions around the world. However, concrete can suffer from deterioration when exposed to certain environmental conditions such as freeze/thaw cycles and external sulfate attack [1].

Exposure to external sulfate attack is one of the main durability concerns in concrete structures exposed to soils, seawater, or groundwater [2,3]. The sulfate attack is typically caused by highly soluble sulfate salts such as magnesium sulfate (MgSO_4_), potassium sulfate (K_2_SO_4_), sodium sulfate (Na_2_SO_4_), and calcium sulfate (CaSO_4_). These sulfate salts react chemically with various phases of the hydrated cement system, causing deterioration of the concrete. The deterioration of concrete due to sulfate attack occurs in the form of spalling, mass loss, softening, expansion, and loss of strength and elastic modulus [4,5,6]. Sulfide oxidation on continents is the main source of seawater sulfate, whereas sulfate reduction and pyrite burial are the main sinks. The concentration of seawater sulfate is regulated by both the atmospheric O_2_ level and the marine redox condition [7]. External sulfate attack is typically a concern when the sulfate concentration in the soil or groundwater is greater than 1000 ppm [8]. Seawater, for example, has a sulfate concentration of approximately 2750 ppm [9].

The impact of external sulfate attack on concrete depends on several factors, including the chemical composition of the cement, the concrete mix design, and the surrounding environment [10,11,12]. The C_3_A content in cement plays a major role in its susceptibility to external sulfate attack as C3A reacts with gypsum to form ettringite. Factors related to concrete mix design, such as the water-to-cement ratio and the use of supplementary cementing materials (SCM), also have a significant impact on the concrete’s resistance to external sulfate attack. A reduction in the water-to-cement ratio and the use of SCMs can improve the permeability of the concrete, thereby reducing the amount of sulfate ions that can penetrate it and cause deterioration. The sulfate ion concentration and the presence of a moisture supply in the environment are two key factors required for the external sulfate attack mechanism to occur. The condition of the concrete may also influence the concrete’s resistance to external attack, such as the existence of cracking.

The two main processes that cause sodium sulfate to attack concrete are the formation of gypsum from the reaction of Na_2_SO_4_ and Ca(OH)_2_ and the formation of ettringite from the reaction of the gypsum with calcium aluminate hydrates. Furthermore, MgSO_4_ breaks down cement by reacting with all cement compounds, including C–S–H, to create gypsum and ettringite [11]. Compared to the other sulfate salts, MgSO_4_ can cause a severe sulfate attack because both magnesium and sulfate ions react with the cement hydration products [13]. In addition, the solubility of MgSO_4_ in water is high, which promotes the movement of these sulfate ions through the concrete pore structure [14]. When the concrete is exposed to an external source of MgSO_4_, two chemical reactions take place, which are summarized below. The first reaction is the chemical reaction of MgSO_4_ with portlandite (Ca(OH)_2_) to form brucite (Mg(OH)_2_) and gypsum (CaSO_4_·2H_2_O), as shown in Equation (1) [15,16]. The increase in the concentration of gypsum in the pore solution promotes the chemical reaction of C_3_A with gypsum to produce ettringite (3CaO·Al_2_O_3_·3CaSO_4_·32H_2_O), as shown in Equation (2) [17,18]. The formation of needle-like ettringite crystals causes the cement paste in concrete to expand [19,20]. The stresses from this expansion can cause concrete to develop microcracks, which may further develop, leading to deterioration of the concrete. In addition, the leaching of calcium compounds (decalcification) from calcium silicate hydrate (C–S–H) due to the reaction of C–S–H with MgSO_4_, which produces brucite, gypsum, and magnesium silicate hydrate (M–S–H), causes the cement matrix to weaken and soften due to the loss of stiffness, as shown in Equation (3) [21,22,23]. Under identical conditions, comparative microanalytical studies of sulfate-resisting Portland cement exposed to Na_2_SO_4_ versus MgSO_4_ solutions revealed that, in comparison to Na_2_SO_4_ exposure, MgSO_4_ exposure produced changes that were generally similar but with more pronounced cracking and material loss, greater ettringite formation, and significantly more decalcification of the C–S–H [24]. The reactions associated with MgSO_4_ attack are represented below as simplified reaction schemes intended to illustrate the principal reaction products. Because C–S–H and M–S–H have variable compositions, Equation (3) in particular should not be interpreted as a fully stoichiometric reaction.

MgSO_4_ + Ca(OH)_2_ + H_2_O → Mg(OH)_2_ + CaSO_4_·2H_2_O (1)

3CaO·Al_2_O_3_ + 3CaSO_4_·2H_2_O + 26H_2_O → 3CaO·Al_2_O_3_·3CaSO_4_·32H_2_O (2)

C–S–H + MgSO_4_ → CaSO_4_·2H_2_O + Mg(OH)_2_ + M–S–H (3)

The above-mentioned reactions between cement hydration products and the sulfate ions typically start at the concrete surface and propagate through the concrete as the sulfate ions absorb and diffuse into the concrete [25]. Although the effect of the external sulfate attack on concrete is well studied in the literature, there is limited research on the effect of external sulfate attack on the concrete surface. Furthermore, we do not clearly understand how the concrete surface’s response to external sulfate attack impacts the overall concrete resistance to external sulfate attack. Considering the recent advancement in microstructure analysis techniques such as the nanoindentation test, evaluating the impact of the external sulfate attack on the concrete surface is warranted [26,27]. Recent literature has further studied the importance of nanoindentation and other micromechanical techniques for understanding sulfate-induced deterioration in cement-based materials. Recent studies have shown that sulfate exposure can modify the micromechanical properties of C–S–H and other hydration products, with changes in indentation modulus and hardness being associated with phase transformation, decalcification, and microcrack development. For example, Hou et al. [28] combined nanoindentation, SEM and XRD with molecular dynamics simulations to demonstrate the degradation of C–S–H micromechanical properties under Na_2_SO_4_ erosion. Yuzhou et al. [29] also employed nanoindentation together with SEM and XRD to examine the microscopic degradation of cement mortar under different sulfate concentrations. Multiscale modeling work by Guan et al. [30] found that nanoindentation-derived phase properties can be linked with macroscopic elastic behavior in sulfate-attacked cement paste [30]. Therefore, it is important to combine nanoindentation with chemical analysis and microstructural evaluations to gain more insight into sulfate-induced deterioration of cementitious pastes. This study aimed to evaluate the effect of MgSO_4_ attack on the surface of a cement paste system. The nanomechanical properties presented by the indentation modulus of the cement paste surface were measured using the nanoindentation test before and after exposure to MgSO_4_. Microstructure characteristics and chemical compositions of the cement paste surface were evaluated using scanning electron microscopy (SEM) with energy-dispersive X-ray spectroscopy (EDX). This study used OPC paste with a w/c ratio of 0.45 as a model cementitious system to isolate the near-surface chemical and micromechanical response to exposure to a 2% wt. MgSO_4_ solution for 7 days, without the additional influence of the aggregates and interfacial transition zones present in concrete.

## 2. Experimental Methods

### 2.1. Materials and Sample Preparation

The cement used in this study was a Type I cement according to ASTM C150 [31]. The cement paste tested in this study had a water-to-cement ratio of 0.45. The prepared cement paste was used to cast 50 mm × 50 mm cubes according to ASTM C109 [32]. After 28 days of moist curing in water, the cube samples were cut to extract disks with a 10 mm square cross-section and 5 mm thickness. From three cubes, three disks (test specimens) were extracted from the center of each cube. A low-speed diamond saw was used to cut and extract the test specimens. Then, the top surface, the testing surface, was exposed to the sulfate solution. Due to the size of the test specimens, sealing was necessary to prevent sulfate propagation through the other faces and to ensure that sulfate attack only occurs at the test surface. Once the epoxy was cured, the test surface was ground and polished until the surface roughness was less than 100 nm.

### 2.2. Exposure and Testing

After the specimen surfaces were polished, the test specimens were immersed in a 2 wt.% MgSO_4_ solution for 7 days at room temperature (23–25 °C), with only the 10 × 10 mm test surface exposed to the solution, corresponding to an exposed area of approximately 100 mm^2^. As sulfate exposure conditions vary in concentration and duration, the findings are specific to the conditions investigated, i.e., a 2% MgSO_4_ concentration. The remaining faces were sealed with epoxy. The experimental data did not record the MgSO_4_ hydration basis, solution-to-specimen volume ratio, solution-renewal history, or pH; therefore, these parameters cannot be reported in this study. The indentation modulus of the test specimen surfaces was measured using a Hysitron Triboindenter (Bruker Corporation, Eden Prairie, MN, USA) equipped with a pyramidal diamond Berkovich tip. Before each nanoindentation test, calibration was performed to eliminate uncertainties and obtain repeatable results. Specimens were prepared for nanoindentation following a multi-step grinding and polishing sequence. Specimens were first impregnated with low-viscosity epoxy resin under vacuum to preserve the pore structure, and then ground sequentially using SiC papers with different grit sizes, followed by polishing using 0.5 µm diamond suspensions on soft polishing cloths, with non-aqueous lubricant used throughout to avoid leaching or further hydration of the paste. Each step was performed for 10 s, and specimens were cleaned ultrasonically in ethanol between steps to remove residual abrasive particles. The test was performed in force-controlled mode with a maximum load of 2000 μN, which was reached in 10 s at a rate of 200 μN/s, followed by a 5 s hold and 10 s of unloading at the same rate. The five-second holding period was used to eliminate any creep effects. The indentation modulus was calculated according to the Oliver and Pharr method by fitting the load–displacement curve. Statistical nanoindentation has previously been used to quantify changes in the nanomechanical properties of C–S–H under environmental and thermal degradation conditions [33]. The modulus obtained using the Oliver–Pharr method [34] is reported throughout this study as the indentation modulus; no conversion to Young’s modulus was performed.

For each test specimen, a total of 300 nanoindentations were conducted, which were divided into three groups of 100 nanoindentations. The spacing between each nanoindentation was 5 µm. A 5 µm spacing was maintained between adjacent indents to limit interaction between measurements, while mapping regions were separated by at least 100 µm. However, individual indents were not assumed to be fully statistically independent because local interactions may depend on indentation depth and microstructural heterogeneity. The chosen blends were examined microstructurally using SEM with EDS Hitachi TM3000 Tabletop SEM (Hitachi High-Tech Corporation, Tokyo, Japan) and backscattered electron detection. The SEM–EDX operating parameters, including the accelerating voltage and beam current, were not retained in the available experimental records and therefore cannot be reliably reported retrospectively. After the nanoindentation test, samples were collected from the specimens’ slightly cracked surfaces. The samples were coated with gold to provide electrical conductivity. Large pores, surface dirt, cracks, polishing scratches, and other glaring preparation flaws were not included. A 10 × 10 indentation array, or 100 indents spread over an area of roughly 100 × 100 μm, with a nominal center-to-center spacing of 5 μm, was used for each mapping region. For the observations to be spatially independent, adjacent mapping sections were spaced at least 100 μm apart. The locations of the indentation maps were chosen to produce a representative sampling of the microstructure while avoiding large pores and obvious phase boundaries unless specifically targeted; 300 nominal indents were performed per specimen. The nanoindentation measurements were used to characterize local mechanical heterogeneity and were not treated as independent material replicates. The reported frequency distributions represent pooled indentation-level data. Since the original data were not retained separately for each specimen, specimen-level mean ± SD values could not be determined retrospectively.

## 3. Results and Discussion

### 3.1. Nanomechanical Properties

**Figure 1:**
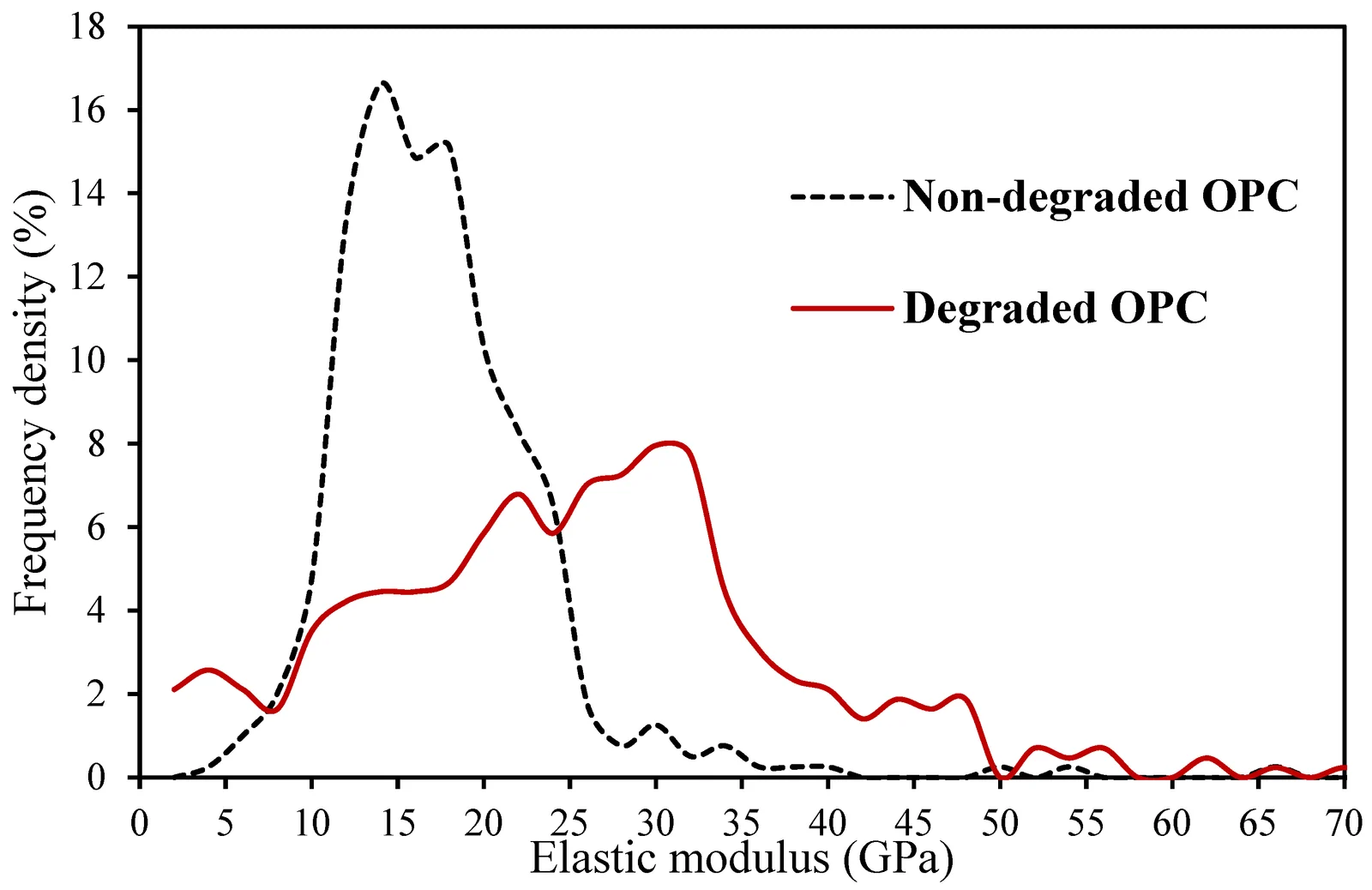
Distributions of nanoindentation moduli before and after sulfate exposure.

**Figure 2:**
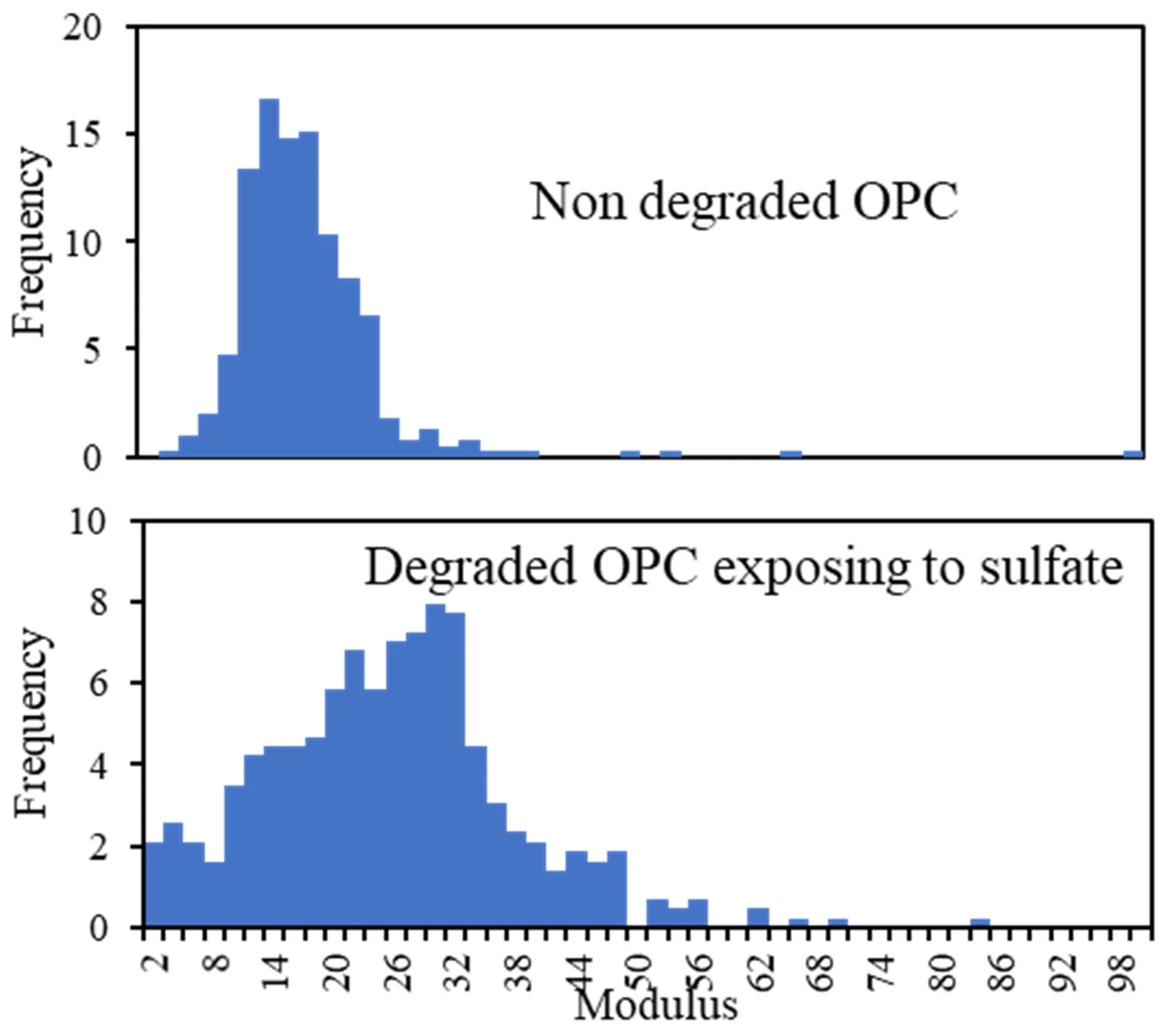
Histogram of indentation modulus.

The results of the nanoindentation test are shown in Figure 1 and Figure 2 and in Table 1. In Figure 1, the test specimen that was not exposed to MgSO_4_ solution is labeled non-degraded OPC, while the test specimen exposed to MgSO_4_ solution is labeled degraded OPC. From Figure 2 and Table 1, it can be observed that the non-degraded OPC test specimen had approximately 3.3% of its indentation points fall from 0 to 9.6 GPa. This value increased to 8.4% when the test specimen was exposed to the MgSO_4_ solution. This increase in the indentation frequency fraction at a low indentation modulus can be attributed to degradation of the cement paste system and the possible formation of microcracks, as confirmed by the image analysis, which is discussed in the following section. Based on the indentation modulus, C–S–H can be classified as low-density C–S–H or high-density C–S–H [35]. Indentation moduli between 9.6 GPa and approximately 32.0 GPa typically represent low-density C–S–H, while values greater than 32.0 GPa typically represent high-density C–S–H. As shown in Table 1, the results show that for the non-degraded OPC test specimen, the indentation frequency fraction of low-density C–S–H was approximately 92.4%. This value decreased to 54.0% after the test specimen was exposed to the MgSO_4_ solution. On the other hand, the indentation frequency fraction with an indentation modulus greater than 32 GPa increased from 4.0% in the non-degraded OPC test specimen to 37.2% in the test specimen exposed to the MgSO_4_ solution. Indentation moduli above 32 GPa are consistent with high-stiffness phases such as high-density C–S–H, unreacted clinker remnants, and/or brucite. Brucite solubility decreases with increasing pH (i.e., with increasing hydroxide ion concentration), consistent with its solubility equilibrium. Due to decalcification or ongoing sulfate/magnesium attack, brucite solubility increases, potentially destabilizing previously precipitated brucite and altering its protective role at the exposed surface. The high pH of the cement paste environment provides chemically favorable conditions for Mg(OH)_2_ formation during MgSO_4_ exposure. Accordingly, the Mg enrichment observed at the exposed surface may be associated with the formation of brucite or other Mg-bearing reaction products. The increased proportion of measurements with indentation moduli above 32 GPa is consistent with the presence of locally dense or high-stiffness regions near the exposed surface. However, because the present study relies on SEM–EDX for chemical characterization, the specific contribution of brucite to the measured modulus cannot be isolated. Therefore, the observed increase in high-modulus measurements is interpreted as being associated with the development of a locally denser Mg-rich reaction layer rather than being attributed exclusively to brucite formation. This finding agrees with the findings of other studies in the literature [36]. It has been reported in the literature that the precipitation of brucite on the surface of the cement paste system can provide a dense protection layer that can decrease the ionic transport into the cement system. This mechanism was reported to be the cause of the reduction in concrete deterioration and expansion when a cement paste system was exposed to a sulfate environment [36].

Based on the modulus of elasticity results from the nanoindentation test, the precipitation of brucite on the surface of the cement paste created a dense layer that caused the proportion of high-modulus measurements to increase substantially in the surface indentation modulus test. This increase in the proportion of high-modulus measurements is consistent with a locally denser microstructure at the cement paste surface, which may be associated with reduced permeability in this region, though this was not directly measured in the present study.

The simultaneous increase in both low- and high-indentation-modulus fractions, accompanied by a decrease in the intermediate fraction, indicates increased microstructural heterogeneity rather than an overall improvement in stiffness. This behavior suggests the coexistence of a stiff Mg-rich surface reaction layer and locally degraded or softened regions following MgSO_4_ exposure.

### 3.2. Microstructural Characterization

**Figure 3:**
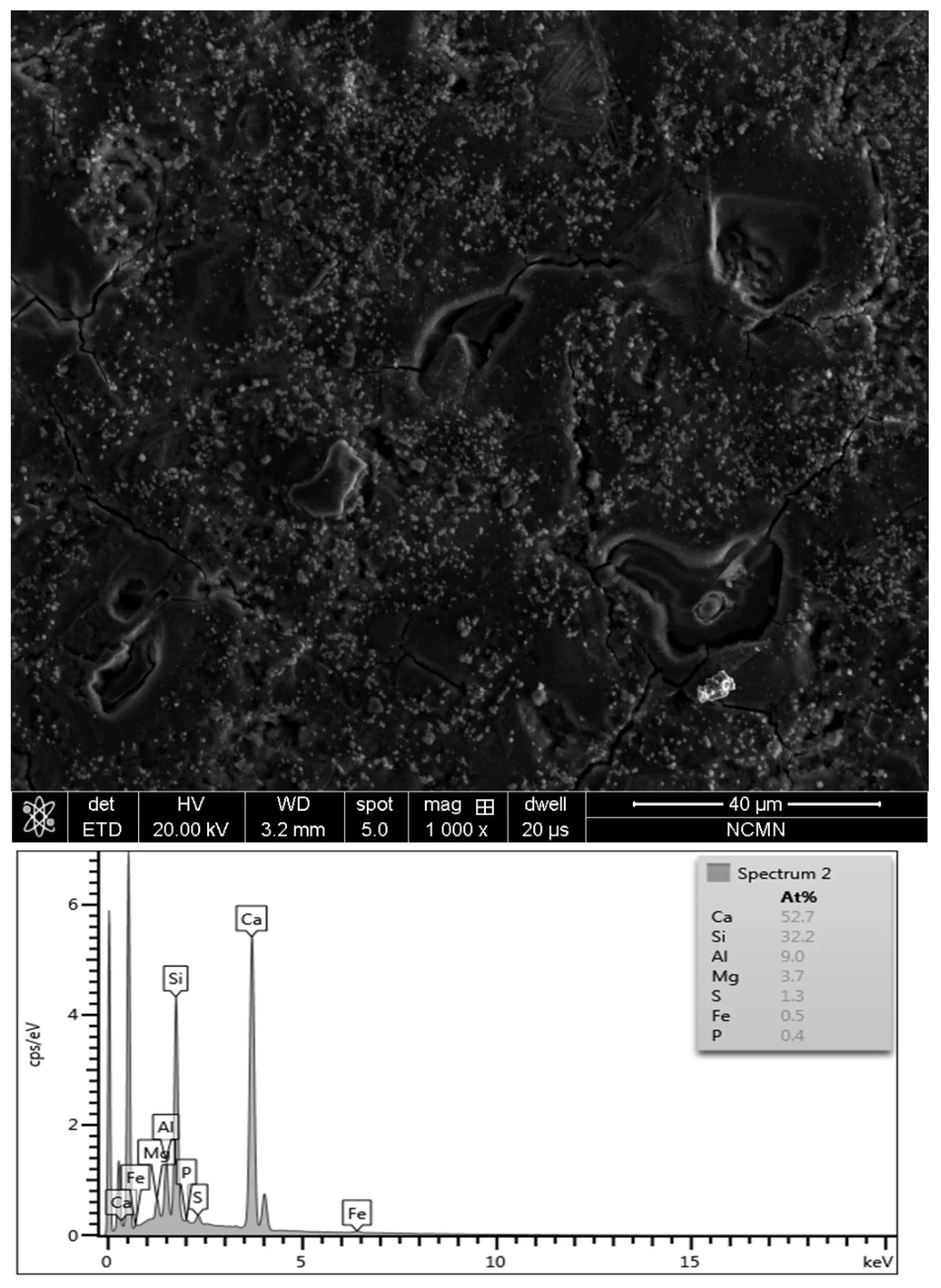
SEM image and EDX analysis of cement paste surface before exposure to MgSO_4_ solution.

The SEM/EDX analysis of the test specimen surface before exposure to the MgSO_4_ solution is shown in Figure 3. As depicted in Figure 3, the SEM image shows a dense micrograph with minimal microcracking on the surface prior to exposure to the MgSO_4_ solution. Microcracks were observed at the surface of MgSO_4_-exposed specimens. Paste expansion was not directly measured in this study, and the expansive phases responsible for the cracking were not conclusively identified or quantified. The production of expansive phases like gypsum and/or ettringite, which are frequently linked to expansive processes in magnesium sulfate attack, is consistent with the cracking. The EDX analysis showed a Ca/Si ratio of 1.64, which is expected for C–S–H produced from Type I cement without any sulfate exposure [37]. Gomes and Oliveira [38] reported that the presence of magnesium hydroxide tends to create voids and microcracks in specimens. Moreover, the appearance of pentagonal plates is likely caused by the sulfated compounds.

**Figure 4:**
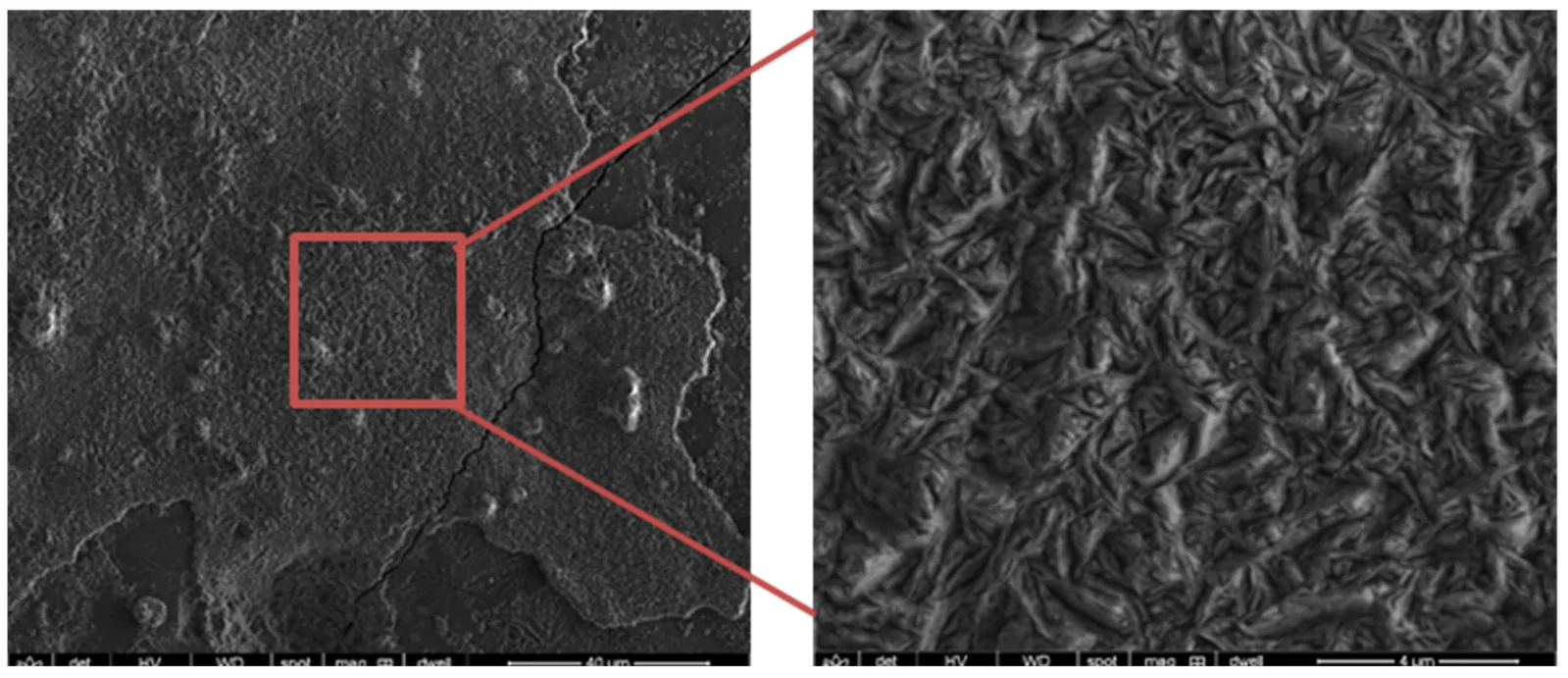
SEM image of test specimen surface exposed to MgSO_4_ solution.

**Figure 5:**
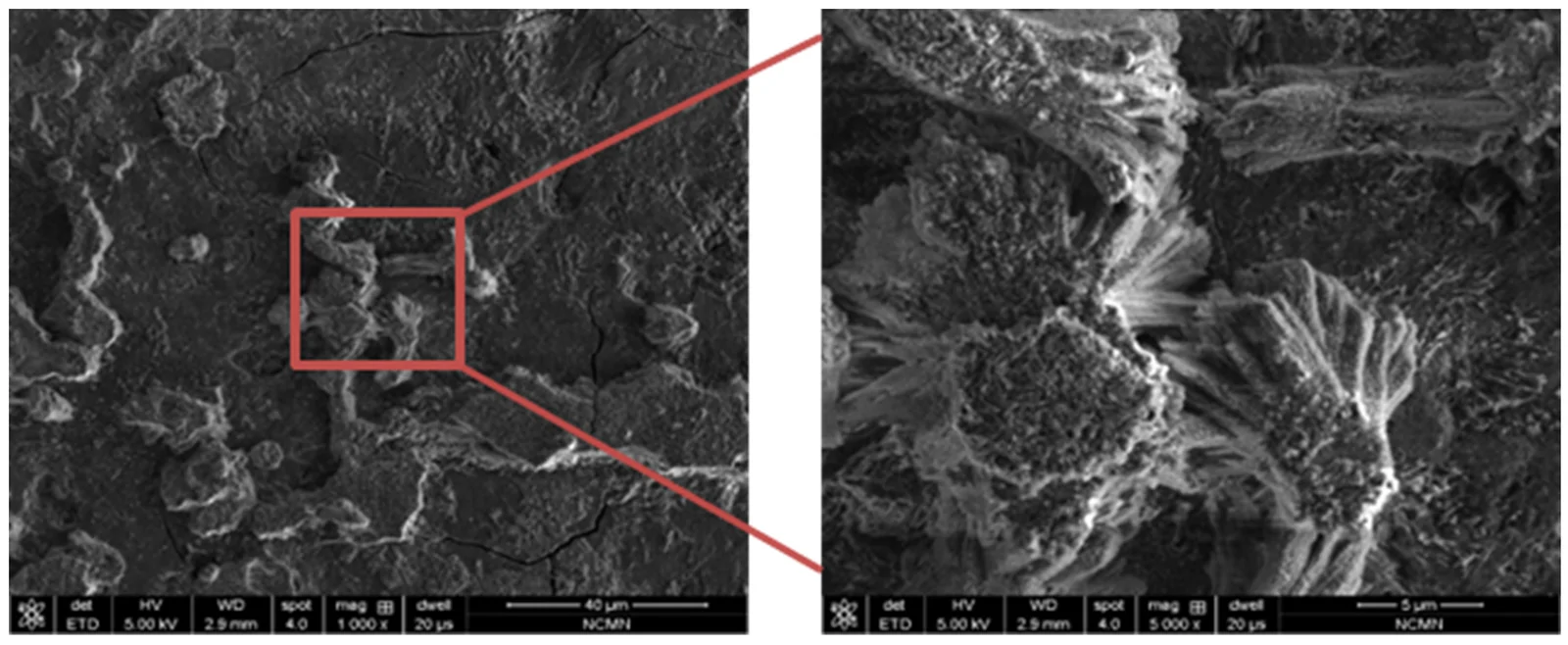
SEM image of test specimen surface exposed to MgSO_4_ solution.

Figure 4 and Figure 5 present the surface of the test specimen after exposure to the MgSO_4_ solution. Figure 4 shows the formation of a dense layer that covers a significant portion of the surface of the test specimen. Figure 4 shows the development of microcracks with a crack width of approximately 1 µm. The observed microcracking may be associated with the formation of expansive reaction products, such as gypsum and/or ettringite, during MgSO_4_ exposure. Considering that the test specimens were subjected to a MgSO_4_ solution for only 7 days, this might not be sufficient to produce enough ettringite to cause paste expansion. The formation of the surface reaction layer has important implications for the transport properties of cementitious materials exposed to aggressive environments. Precipitation of Mg(OH)_2_ (brucite), if formed under the present exposure conditions, could partially block capillary pores and contribute to densification of the outer paste layer. However, the present SEM–EDX observations alone cannot confirm that the Mg-rich layer consists exclusively of brucite [21]. A study by Taheri et al. [39] revealed that, instead of creating a consistent Mg(OH)_2_ barrier, chemically and structurally heterogeneous layers—MgO-rich inner parts, porous mixed MgO/Mg(OH)_2_ outer regions, and layers with hydration, cracking, and varying degrees of brucite production—were formed. Consequently, the initial ingress of sulfate ions may be inhibited despite the ongoing chemical reactions occurring beneath the surface. Similar observations have been reported in previous studies, where the low solubility of Mg(OH)_2_ promoted the formation of a relatively dense surface layer that temporarily reduced ion diffusion [40]. The precipitation of Mg(OH)_2_ may occur simultaneously with the consumption of portlandite and progressive decalcification of calcium silicate hydrate (C–S–H). The coexistence of a dense reaction layer and chemically weakened hydration products demonstrates the complex nature of MgSO_4_ attack during its early stages.

This was confirmed using SEM-EDX analysis, which showed limited morphology, likely with ettringite formation at the surface of the test specimen (Figure 5). Therefore, the microcracking observed in Figure 4 may be associated with the formation of gypsum and/or ettringite; however, their direct contribution to cracking cannot be confirmed from the present SEM–EDX observations. The microcracks observed are significant because they represent the transition from purely chemical deterioration to integration of physicochemical degradation. Although the measured crack widths remained relatively small after 7 days of exposure, these defects can substantially influence long-term durability by providing preferential pathways for the ingress of aggressive ions. Continued sulfate penetration through these microcracks is expected to accelerate the dissolution of hydration products and promote further precipitation of expansive reaction products. Consequently, the microcracks found on the cement paste depict an early indicator of progressive deterioration rather than evidence of severe structural damage. Using SEM-EDX analysis to detect these early defects can complement nanoindentation measurements by linking the conventional reductions in mechanical properties of the cement paste with detectable microstructural changes. This multiscale interpretation agrees with recent findings that showed that nanoindentation is capable of capturing localized mechanical deterioration attributed to sulfate-induced changes in microstructural behavior and hydration products [28,41].

### 3.3. Chemical Analysis

**Figure 6:**
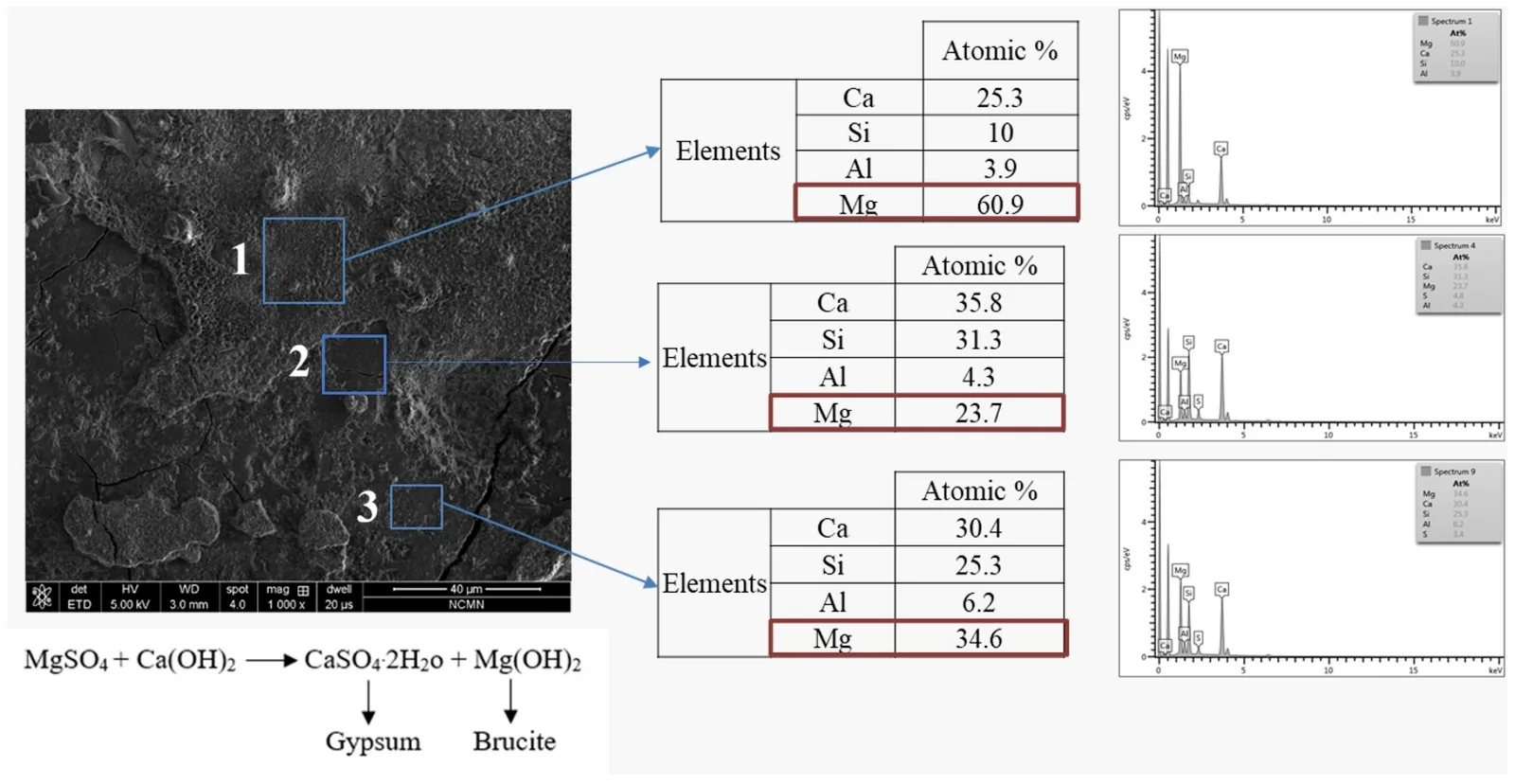
EDX test results for OPC exposed to sulfate.

**Figure 7:**
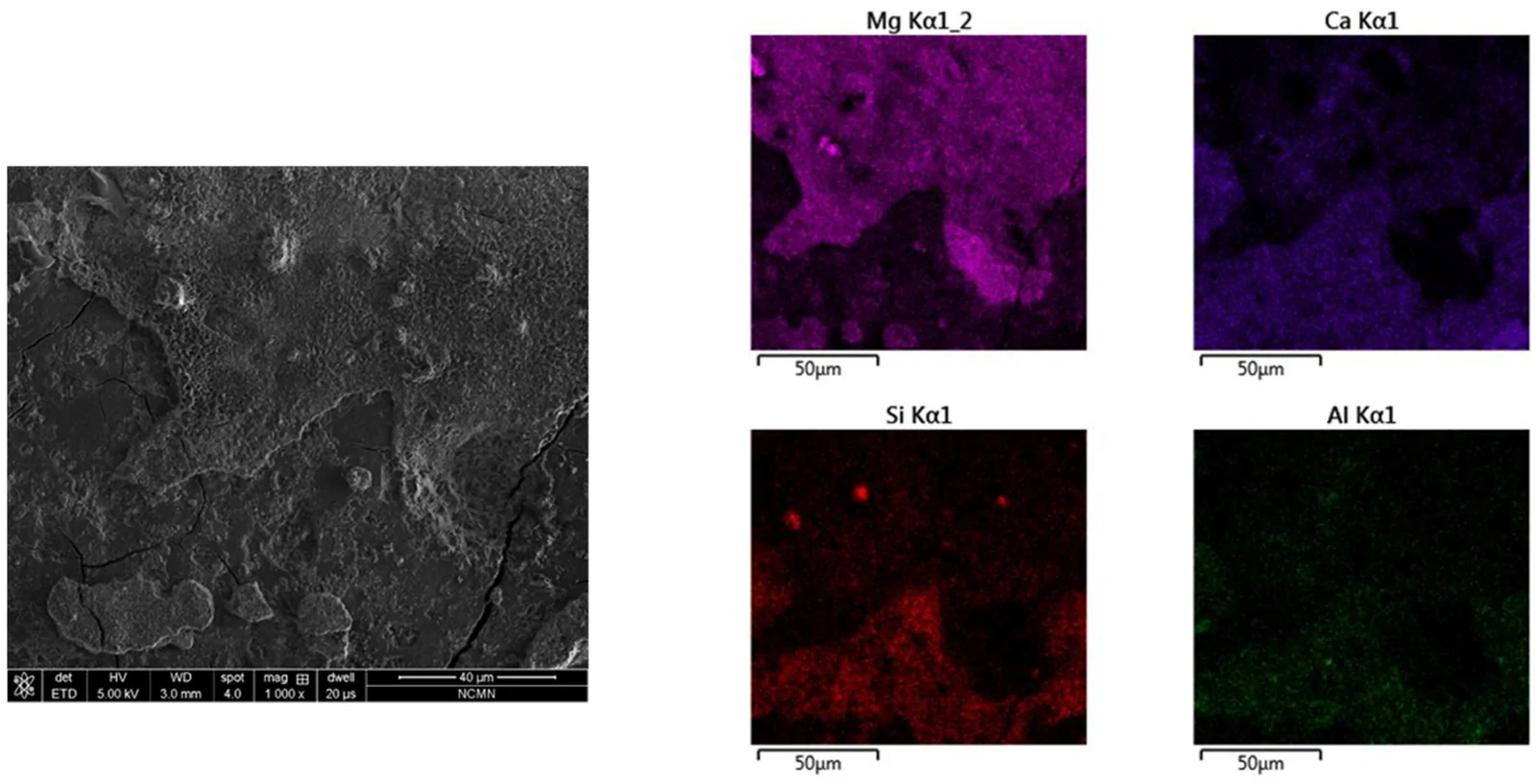
Chemical mapping after exposure to MgSO_4_.

Chemical analysis was performed to characterize the elemental composition and assess the hydration products in the cementitious systems. The spectra (Figure 6 and Figure 7) confirm the presence of Ca, Si, Al, and Mg, indicating the formation of calcium–silicate-hydrate-based reaction products. The atomic ratios obtained from selected regions (Table 2) provide insight into the evolution of the hydration process and the formation of C–S–H products. The elemental compositions obtained from EDX shown in Table 2 are reported as atomic percentages. The locations of EDX Areas 1–3 are identified in Figure 6 and correspond to the elemental compositions reported in Table 2.

The variations in elemental composition detected among the analyzed regions indicate that the attack by MgSO_4_ was spatially heterogeneous. Rather than occurring uniformly across the exposed surface, the deterioration process progressed through specified chemical reactions controlled by the distribution of hydration products and pore connectivity. Areas with high Mg concentrations indicate extensive interaction of Mg^2+^ with the cement matrix. Under the alkaline pore-solution conditions, this Mg enrichment is consistent with the precipitation of Mg(OH)_2_ (brucite) and/or other Mg-bearing reaction products rather than precipitation of MgSO_4_ itself. On the contrary, areas exhibiting lower calcium concentrations indicate progressive decalcification of the C–S–H matrix. This chemical heterogeneity explains the variability observed in the nanoindentation measurements, where both low-modulus and high-modulus regions coexist within the same specimen.

The effective interaction volume of EDX under the measurement conditions is expected to be in the order of micrometers and depends on factors such as accelerating voltage, material composition, and density. Therefore, the measured elemental composition represents a near-surface interaction volume rather than a fixed penetration depth. The EDX interaction volume analysis samples a near-surface region and therefore the measured elemental composition represents the analyzed interaction volume rather than a uniquely identified mineral phase. The EDX analysis revealed substantial Mg enrichment within the dense surface layer, which is consistent with brucite formation. The EDX analysis revealed the presence of Ca, Si, Al, and Mg on the cement paste surface after exposure to MgSO_4_, with substantial spatial variation in the elemental composition (Figure 6 and Table 2 and the chemical mapping (Figure 7). The Mg contents of the analyzed regions ranged from 23.7% to 60.9%, indicating significant Mg enrichment following sulfate exposure. This Mg enrichment, together with the known reaction between MgSO_4_ and portlandite, is consistent with the formation of Mg(OH)_2_ (brucite) or other Mg-bearing reaction products. However, EDX provides the elemental composition but cannot independently identify the crystal structure or mineralogical phase. Therefore, the dense surface feature observed in the SEM images is described here as a Mg-rich surface layer consistent with brucite formation, rather than being conclusively identified as brucite.

The lower Ca contents observed in some Mg-enriched regions may indicate calcium depletion associated with MgSO_4_-induced decalcification and modification of the cement paste matrix. The coexistence of Mg enrichment and Ca depletion supports the occurrence of chemical alteration at the exposed surface. The increased fraction of high-modulus nanoindentation measurements may be associated with the development of this relatively dense Mg-rich surface region. Nevertheless, the specific contribution of brucite to the measured indentation modulus cannot be isolated from the present SEM–EDX and nanoindentation results. Phase-sensitive characterization such as XRD, Raman spectroscopy, or FTIR would be required to conclusively identify the mineralogical phases within the Mg-rich layer. This finding agrees with recent findings of Hou et al. [28], where they found reduced indentation modulus and hardness of C–S–H during the progression of sulfate erosion, with the deterioration linked to the formation of ettringite, gypsum and microcracks. Sun et al. [41] also found in their recent investigations that sulfate exposure yields noticeable changes in the micro-scale mechanical properties of cement-based materials, which are associated with microstructural and chemical deterioration. The results of this study are also consistent with the distinct nanomechanical behavior of low-density and high-density C–S–H observed in nanoindentation assessments performed by Constantinides and Ulm [42] and Constantinides and Ulm [43].

The chemical analysis also confirms that the deterioration mechanism cannot be explained solely by sulfate attack. In contrast to Na_2_SO_4_, MgSO_4_ actively changes the chemistry of the primary binding phase through ion exchange and calcium leaching. This additional reaction pathway accelerates the degradation of C–S–H and promotes the formation of magnesium-bearing products with inferior binding characteristics. Consequently, the combined presence of magnesium enrichment and calcium depletion provides direct evidence that the observed mechanical and microstructural changes originate from chemically induced modification of the cement matrix. This linkage between the SEM analysis, EDX results and nanoindentation measurements shows that these methodologies supplement each other in describing the advancement of early MgSO_4_ deterioration in cement paste.

## 4. Conclusions

This study evaluated the changes to the surface of a cement paste system after exposure to a 2 wt.% MgSO_4_ solution for 7 days. Nanomechanical properties were assessed using nanoindentation tests, and microstructural and chemical analyses of the cement paste surface were conducted before and after exposure to the 2 wt.% MgSO_4_ solution for 7 days. Based on the results of this study, the following conclusions can be drawn:

1. The nanoindentation results showed that the fraction of measurements with an indentation modulus greater than 32 GPa increased from 4.0% before exposure to 37.2% after 7 days of MgSO_4_ exposure, corresponding to an increase of 33.20 percentage points. Meanwhile, the percentage of measurements below 9.6 GPa increased from 3.3% to 8.4%. This indicates a greater occurrence of local high-stiffness regions rather than a 33% increase in the indentation modulus itself or an increase in degraded regions following sulfate exposure.
2. The SEM–EDX analysis revealed a dense Mg-rich surface layer approximately 1–2 µm thick after 7 days of MgSO_4_ exposure. The observed Mg enrichment is consistent with Mg(OH)_2_ (brucite) formation; however, EDX alone cannot conclusively identify the mineralogical phase.
3. The increased fraction of high-indentation-modulus values is consistent with the development of a stiff Mg-rich surface reaction layer, potentially containing brucite, which is supported by the SEM–EDX observations. However, the specific contribution of brucite cannot be conclusively established.
4. The surface of the tested cement paste system showed signs of degradation after exposure to the MgSO_4_ solution for 7 days. Although the exposure to the MgSO_4_ solution was limited to 7 days, the development of microcracks and C–S–H degradation indicates that the treatment causes localized near-surface alterations rather than severe bulk deterioration in the cement paste matrix.
5. The present findings specifically represent the early stage of MgSO_4_ attack after 7 days of exposure, with Mg-rich surface-layer development, microcracking, and changes in indentation modulus being observed. The development of microcracks and C–S–H degradation indicates the severity of this type of sulfate attack on the cement paste matrix. Further deterioration with prolonged exposure can be expected; however, its extent and evolution cannot be established from the present short-term study and require longer-term investigation.
6. A limitation of this study is that EDX provides elemental compositions but does not directly identify mineral phases. Therefore, although the observed Mg-rich surface layer is chemically consistent with brucite, other Mg-bearing phases cannot be excluded. Phase-sensitive techniques such as XRD, Raman, or FTIR would be required for definitive phase identification. Another limitation of this study is that the nanoindentation data were analyzed as pooled frequency distributions rather than at the specimen level. Therefore, between-specimen variability and mean ± SD values could not be determined. Future studies should employ specimen-level analysis to improve statistical robustness.

## Figures and Tables

**Table 1:** Pooled frequency distribution of nanoindentation modulus measurements before and after MgSO_4_ exposure.

	Frequency of Occurrence (%)	
Indentation Modulus (GPa)	Non-Degraded	Degraded
4.75 ± 4.7	3.27	8.43
20.3 ± 12.6	92.44	54.09
>32	4.03	37.23

**Table 2:** EDX elemental composition (atomic %) and atomic ratios.

Scan Area	Elements and Elemental Ratios						
	Si (%)	Ca (%)	Al (%)	Mg (%)	Ca/Si	Si/Al	Ca/(Si + Al)
1	10	25.3	3.9	60.9	2.53	2.56	1.82
2	31.3	35.8	4.3	23.7	1.14	7.28	1.03
3	25.3	30.4	6.2	34.6	1.20	4.08	0.97

## Data Availability

The original contributions presented in this study are included in the article. Further inquiries can be directed to the corresponding author.
